# Gut Microbial Metabolite Butyrate and Its Therapeutic Role in Inflammatory Bowel Disease: A Literature Review

**DOI:** 10.3390/nu15102275

**Published:** 2023-05-11

**Authors:** Neeraja Recharla, Ramasatyaveni Geesala, Xuan-Zheng Shi

**Affiliations:** Department of Internal Medicine, The University of Texas Medical Branch, 301 University Blvd, 4.106 Basic Science Building, Galveston, TX 77555-0655, USA; nerechar@utmb.edu (N.R.); rageesal@utmb.edu (R.G.)

**Keywords:** butyrate, inflammatory bowel disease, gut microbiota, microbial metabolites, nutrients, gut homeostasis, immune responses, T-cells

## Abstract

**Background and objective**: Inflammatory bowel disease (IBD), including Crohn’s disease and ulcerative colitis, is a chronic inflammatory disorder characterized by aberrant immune responses and compromised barrier function in the gastrointestinal tract. IBD is associated with altered gut microbiota and their metabolites in the colon. Butyrate, a gut microbial metabolite, plays a crucial role in regulating immune function, epithelial barrier function, and intestinal homeostasis. In this review, we aim to present an overview of butyrate synthesis and metabolism and the mechanism of action of butyrate in maintaining intestinal homeostasis and to discuss the therapeutic implications of butyrate in IBD. **Methods:** We searched the literature up to March 2023 through PubMed, Web of Science, and other sources using search terms such as butyrate, inflammation, IBD, Crohn’s disease, and ulcerative colitis. Clinical studies in patients and preclinical studies in rodent models of IBD were included in the summary of the therapeutic implications of butyrate. **Results:** Research in the last two decades has shown the beneficial effects of butyrate on gut immune function and epithelial barrier function. Most of the preclinical and clinical studies have shown the positive effect of butyrate oral supplements in reducing inflammation and maintaining remission in colitis animal models and IBD patients. However, butyrate enema showed mixed effects. Butyrogenic diets, including germinated barley foodstuff and oat bran, are found to increase fecal butyrate concentrations and reduce the disease activity index in both animal models and IBD patients. **Conclusions:** The current literature suggests that butyrate is a potential add-on therapy to reduce inflammation and maintain IBD remission. Further clinical studies are needed to determine if butyrate administration alone is an effective therapeutic treatment for IBD.

## 1. Introduction

Inflammatory bowel disease (IBD) is characterized by chronic intestinal inflammation in the gastrointestinal (GI) tract and includes Crohn’s disease (CD) and ulcerative colitis (UC). Although both CD and UC present with chronic inflammation, they differ in many aspects such as location, distribution, and depth of inflammation, and complications, and rectal involvement (Table 1). The exact cause of IBD is still not well understood, but the pathogenesis is interlinked with genetic factors, abnormal immune reactivity, microbiota dysbiosis, diet, and environmental factors being involved. The dynamic balance between commensal microflora and host defensive responses in the intestine plays a key role in the initiation and chronic progression of IBD [1]. Disturbed immune function and epithelial barrier integrity are the major features of IBD.

Since the pathogenic mechanisms of CD and UC remain unknown, IBD is not curable. Current therapies for IBD, including corticosteroids, immunomodulators, and biologics, are designed to induce remission [2]. However, patient response to the treatments decreases over time, and relapses occur frequently. Moreover, the side effects of these treatments are significant, and sometimes intolerable to patients. It is important to identify novel therapeutic targets and discover effective and safe treatments for IBD patients. Short-chain fatty acids (SCFAs) are the most abundant microbial metabolites in the intestine and provide 60–70% of the energy needs for colonocytes [3]. Specifically, butyrate is the major fuel source for the epithelial cells and has gained more attention than any other SCFAs as it regulates intestinal homeostasis and maintains epithelial integrity. A reduced number of butyrate-producing bacteria and lowered butyrate concentration have been found in IBD [4,5]. As butyrate is shown to modulate immune function and intestinal barrier function, it is considered a therapeutic target in the treatment of IBD. In this review, we discuss the production and metabolism of butyrate and the therapeutic implications of butyrate in IBD.

The literature for this review was searched up to March 2023 from PubMed, Web of Science, and other sources using search terms such as butyrate, inflammation, IBD, Crohn’s disease, and ulcerative colitis. Studies on human trials and animal models were included to extract data for summarizing the therapeutic implications of butyrate. The relevant studies and their reported outcomes were analyzed and discussed with references to emphasize possible mechanisms of action. This review only includes papers published in English.

## 2. Gut Microbiota and Metabolites

The human gastrointestinal tract harbors a complex and diverse microbial population termed gut microbiota. The gut microbiota comprises trillions of microbes, including archaea, bacteria, fungi, and viruses. Many bacteria, particularly anaerobic bacteria, colonize the intestinal tract in a symbiotic relationship which plays a critical role in maintaining the intestinal homeostasis of the host. The high-throughput DNA sequencing technology has enhanced our understanding of gut microbiota without the need for microbial culturing. More than 1000 bacterial species colonized the human gastrointestinal tract, especially in the colon. Most of these bacterial species belong to two major phyla: *Firmicutes* and *Bacteroidetes* [6].

**Figure 1:**
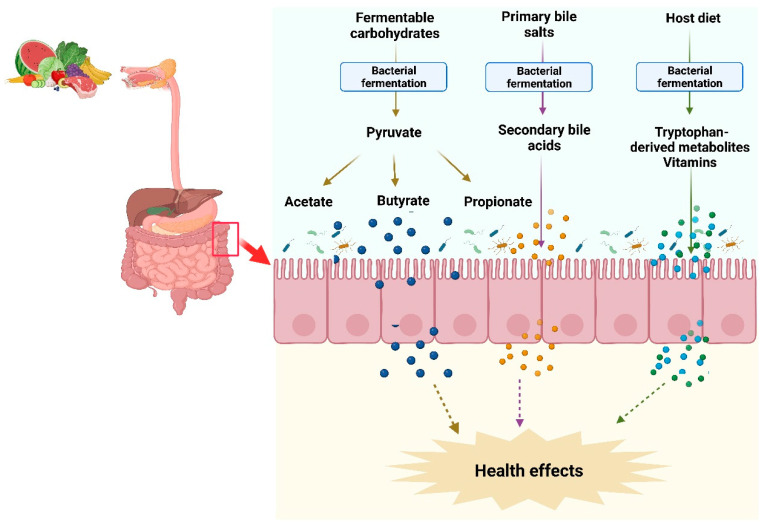
Synthesis of microbial metabolites in the intestine. Commensal bacteria in the intestine utilize nondigested fermentable carbohydrates and proteins from the host-ingested diet and produce SCFAs and vitamins. Likewise, gut bacteria transform nonabsorbed primary bile salts into secondary bile acids. These microbial metabolites modulate the host physiological functions and provide health benefits.

The gut microbiota produces a wide range of metabolites, including SCFAs, polyamines, vitamins, tryptophan-derived metabolites, and secondary bile acids, using exogenous undigested dietary substrates and endogenous compounds [7]. These metabolites can be classified into three types: (1) Metabolites produced by the microbial transformation of dietary components or drugs such as compound K; (2) Metabolites produced from host secretions that are modified by gut microbiota such as secondary bile acids; and (3) Metabolites synthesized by gut microbiota from diet components such as SCFAs [8]. These metabolites may also serve as nutrients or substrates for other bacterial species in the colon to further produce metabolites via interspecies cross-feeding interactions [9]. The microbial metabolites can be both beneficial and toxic to the host (Figure 1).

The primary bile acids, cholic acid, and chenodeoxycholic acids are synthesized from cholesterol and conjugated to glycine or taurine in the liver then stored in the gallbladder and released into the intestine to facilitate dietary-fats emulsification, digestion, and absorption in the small intestine. The remaining bile acids are absorbed in the terminal ileum and reached the liver through enterohepatic circulation [10]. The escaped bile salts during enterohepatic circulation become substrates for gut microbial metabolism, including deconjugation, oxidation, epimerization, and dehydroxylation. The bacteria genera including *Clostridium*, *Bifidobacterium*, *Bacteroides*, *Listeria*, and *Lactobacillus* are involved in the deconjugation of bile acids. *Bacteroides*, *Eggerthella*, *Escherichia*, *Clostridium*, *Ruminococcus*, and *Peptostreptococcus* are involved in oxidation and epimerization [11]. The intestinal bacteria *Clostridium* and *Eubacterium* genera transform cholic acid and chenodeoxycholic acid into deoxycholic acid and lithocholic acid, respectively, by dihydroxylation using hydroxysteroid dehydrogenase enzymes [11,12,13]. Undigested dietary proteins enter the colon and serve as substrate for gut-microbial metabolism. Tryptophan is an essential amino acid consumed in the diet. Undigested or escaped tryptophan is fermented by colonic bacteria, producing various metabolites, indole, indoleacetic acid, indole-3-lactate, and indole-3-propionate through direct tryptophan transformation pathway [14]. Indole-producing bacteria, such as *Acinetobacter oleivorans*, *Vibrio cholera*, *Escherichia coli*, *Pseudomonas chlororaphis*, and *Synbiobacterium thermophilus*, produce indole from tryptophan [15].

SCFAs, including acetic, propionic, and butyric acids, are a group of carboxylic acids that consist of lesser than six carbon atoms. SCFAs are derived from the fermentation of nondigestible carbohydrates in the proximal colon and by proteolytic fermentation in the distal colon. SCFAs can be formed from fermentable carbohydrates through the glycolytic pathway and the pentose phosphate pathways by microbial fermentation [16]. Butyrate is mainly produced from species of the *Firmicutes* phylum, including *Roseburia* species, *Faecalibacterium prausnitzii*, and *Eubacterium rectale*, whereas acetate and propionates are produced from the species of the *Bacteroidetes* phylum [17,18]. The production of SCFAs in the intestine is substrate dependent. About 300 to 600 mmol of SCFAs are produced in the human intestine per day and only a small amount of SCFAs (~10 mmol) are excreted through fecal excretion. The remaining SCFAs are rapidly absorbed by the host epithelial cells via passive diffusion or active transport [19,20].

## 3. Butyrate Production, Absorption, and Metabolism

**Figure 2:**
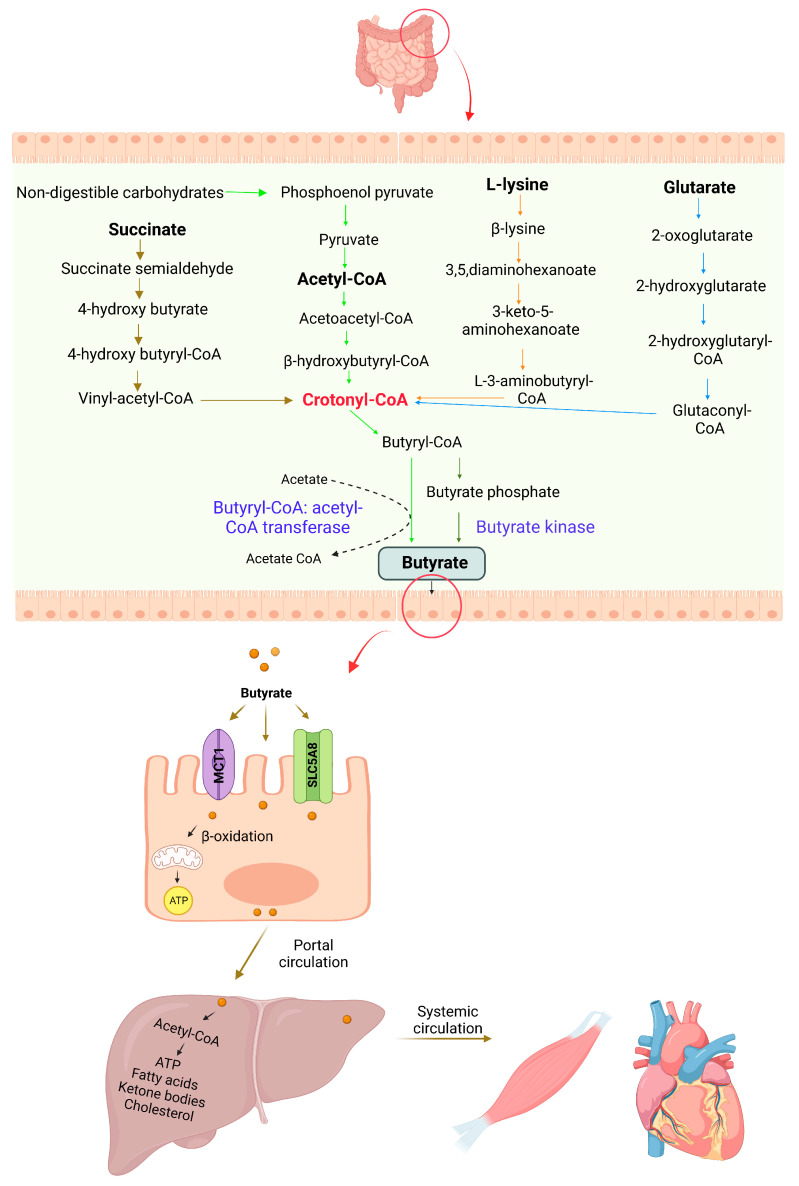
Schematic representation of pathways involved in butyrate production, absorption, and metabolism. Butyrate is synthesized by intestinal bacteria via four pathways from nondigestible carbohydrates, succinate, L-lysate, and glutarate. It is taken-up and metabolized by the colonic epithelial cells. Low levels of butyrate enter into the liver and regulate fatty acid metabolism. Small amounts of butyrate enter into the systemic circulation and may reach other tissues.

Gut microbiota produces butyrate from acetyl-CoA, lysine, glutarate, or succinate pathways in the colon [21]. Various bacterial species in the human intestine generate enzymes that can synthesize butyrate from complex fermentable substrates. The predominant butyrogenic bacterial species, including *Faecalibacterium prausnitzii*, *Clostridium* spp., *Eubacterium* spp., and *Roseburia* spp., are from two clusters (*Clostridium* clusters IV and XIVa) in the *Firmicutes* phylum and the *Clostridiales* order [22,23]. Most luminal butyrate is synthesized from nondigestible carbohydrates via the acetyl-CoA pathway (Figure 2). In the first step, nondigestible carbohydrates are catabolized into pyruvate through the pentose phosphate pathway or Embden–Meyerhof–Parnas pathway. Pyruvate can be converted into acetyl-CoA, which is further broken down into butyryl-CoA. In the final step, butyryl-CoA can be converted into butyrate by butyryl-CoA: acetyl-CoA transferase or phosphorylated to butyryl-phosphate through phospho-transbutyrylase and then subsequently converted to butyrate through butyrate kinase [24,25,26]. Acetate is required to produce butyrate via butyryl-CoA: acetyl-CoA transferase through cross-feeding microbial reactions. Butyrate is produced by cross-feeding interactions between acetate-producing *Bifidobacterium* spp. and acetate-utilizing *Faecalibacterium prausnitzii* [23]. Moreover, the metabolite cross-feeding within the microbial community plays a key role in maintaining the diversity of the gut-microbial ecosystem [27]. In the succinate pathway, butyrogenic bacteria convert succinate to crotonyl-CoA, which is subsequently converted into butyrate. Crotonyl-CoA is the common butyrate precursor in L-lysine and glutarate pathways (Figure 2). 

Gut-microbiome-derived butyrate is taken up rapidly by colonocytes through passive nonionic diffusion or active carrier-mediated transport [28]. The ionized form of butyrate is transported across the apical surface of intestinal epithelial cells through active transport mediated by H^+^-monocarboxylate transporter-1 (MCT1) and Na^+^-coupled monocarboxylate transporter-1 (SMCT1). Solute carrier family 5 member 8 (SLC5A8) is one of the major SMCT1 transporters of butyrate across the colonocytes [29]. The gene expression levels of SLC5A8 are abundant in the apical membrane of the colon and ileum. On the basolateral membrane, butyrate is transported through the carrier-mediated bicarbonate exchange system [30]. Butyrate predominantly presents in the anionic form in the colon due to colonic luminal pH conditions. Thus, it requires carrier-mediated transportation for cellular entry.

The absorbed butyrate is metabolized in the intestinal epithelial cells, liver cells, and other tissues and cells [31]. In the epithelial cells, butyrate is transformed into acetyl-CoA and enters the tricarboxylic acid (TCA) cycle in the mitochondria to produce ATP, which is consumed by the colon epithelial cells. The portion of butyrate which is not utilized by epithelial cells can reach the liver via portal circulation, where it is metabolized into acetyl-CoA and becomes a substrate for fatty acids, cholesterol, and ketone bodies by hepatocytes [22,32]. The plasma concentration of butyrate is very low compared to colonic levels, only 2% of butyrate enters systemic circulation, being utilized by other tissues and cells [32]. The remaining SCFAs, including butyrate, are excreted through the lungs and urine. 

## 4. Role and Mechanisms of Butyrate in the Regulation of Barrier Function and Immune Response

The single layer of intestinal epithelium serves as a barrier between the host and its external environment that controls the interaction between the luminal contents and the internal milieu of the body. The intestinal epithelial monolayer contains several types of specialized cells: (1) enterocytes, for absorption of nutrients; (2) goblet cells, producing secretory and gel-forming mucins which are glycosylated proteins that form polymeric nets called mucus layer, a physical barrier between intestinal bacteria and epithelial cells; (3) enteroendocrine cells, secreting various hormones regulating digestive function; (4) Paneth cells, residing at crypt base and secreting antimicrobial peptides such as lysozyme, defensins, and cryptidins; (5) microfold cells (M cells), sampling antigens from the lumen to subepithelium; and (6) tuft cells, for chemosensing function in the epithelium [33,34]. These epithelial cells are connected by intercellular desmosomes, tight junctions (TJs), and adherent junctions (AJs), which create a physical barrier for luminal contents of the gut and regulate epithelial permeability. TJs are a complex network formed by transmembrane proteins such as claudins, occludin, tricellulin, and junctional adhesion molecules and cytosolic scaffold proteins such as zonulae occludens (ZO) and cingulin [35,36]. Both TJs and AJs are connected to the actin cytoskeleton and form an apical junction complex. On the basal side, epithelial cells are connected by hemidesmosomes.

The intestinal epithelium lies between the commensal organisms in the gut lumen and the immune cells in lamina propria. The complex immune interactions between commensal microflora, the epithelial layer, and the subepithelial immune cells maintain homeostasis under normal conditions. Lamina propria contains the gut-associated lymphoid tissue (GALT) which is comprised of Peyer’s patches, a group of lymphoid follicles containing several immune cells, such as specialized M cells, dendritic cells, T cells, B cells, intraepithelial lymphocytes, and macrophages [37]. The dendritic cells (DCs) from lamina propria sample the luminal food and microbial antigens by extending their dendrites between epithelial cells and transport to antigen-presenting cells (APCs) in GALT [38,39]. Upon activation, GALT performs effector immune functions by activating immune cells to produce specific cytokines from T cells and immunoglobulins from B cells. Antigens in the gut lumen can be taken up by specialized M cells and delivered to DCs for effector functions in the Peyer’s patches [40]. Intestinal epithelial cells themselves can also act as dynamic sensors by pattern recognition molecule receptors (PRRs) such as toll-like receptors (TLRs) and nucleotide-binding oligomerization domain (NOD)-like receptors (NLRs) to sense pathogen-associated molecular patterns.

**Figure 3:**
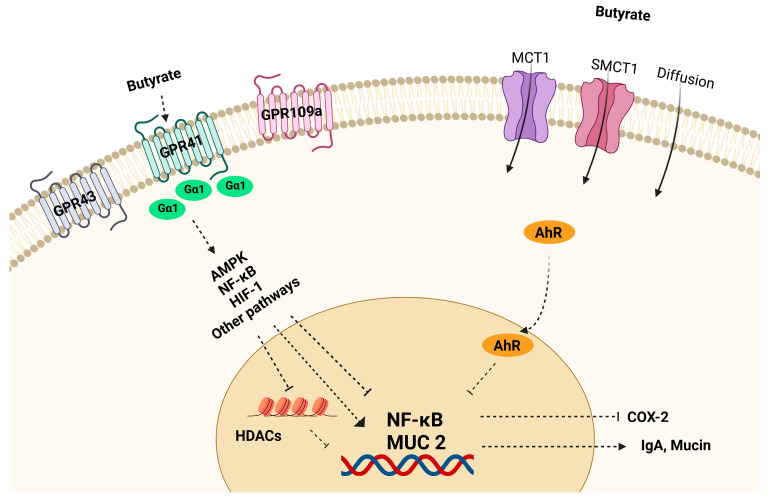
Schematic overview of butyrate transport and cellular mechanisms. Butyrate is absorbed by intestinal epithelial cells via active transport mediated by MCT1 and SMCT1 transporters or via passive diffusion. Butyrate activates GPRs and couples to G proteins to interact with downstream effectors such as HDACs to reduce inflammation.

Gut microflora and their metabolites play a major role in maintaining epithelial barrier function and immune homeostasis. Among the microbial metabolites, butyrate involves a number of signaling pathways in the gut immune cells and epithelial cells for the restoration of impaired colonic barrier function and gut homeostasis (Figure 3). The pathophysiology of IBD involves both epithelial barrier dysfunction and abnormal immune-cell activation. Changes in TJs structure, downregulation of claudin proteins, and upregulation of pore-forming claudin-2 were observed in both CD and UC conditions [34]. Since 2007, butyrate was found to enhance the intestinal barrier function by facilitating tight junction assembly via activation of AMPK, Akt, and other signaling pathways in a dose-dependent manner as shown in studies with transepithelial electrical resistance (TEER) and fluorescein isothiocyanate-dextran (FITC-dextran) permeability assays in in vitro settings [41,42,43]. Marinelli et al. [44] demonstrated that butyrate regulates the epithelial barrier function by acting as a signaling molecule for cell-surface G-protein-coupled receptors (GPRs) and nuclear factors (NFs). Indeed, butyrate was found to induce T cell-independent IgA secretion in the colon via activation of GPR41 (free fatty acid receptor 3, FFAR3) and GPR109A (hydrocarboxylic acid receptor 2, HCAR2), and inhibition of histone deacetylase (HDAC) to restore epithelial barrier function under inflammatory conditions [45]. Studies also explored the effect of butyrate on claudins expression. Zheng et al. [46] reported that butyrate promotes epithelial barrier function through interleukin-10 receptor α-subunit (IL-10RA)-dependent repression of claudin-2 TJ protein. Wang et al. [47] demonstrated that butyrate treatment improved epithelial barrier function via the upregulation of claudin-1 transcription by facilitating the interaction between specific motifs in the claudin-1 promoter region and SP1 transcription factor. Moreover, butyrate enhances mucin secretion and protects epithelial cells by inducing MUC2 gene expression via AP-1 and acetylation/methylation of histones at the MUC2 promoter in intestinal epithelial goblet cells [48]. Hypoxia-inducible factor 1 (HIF-1)-dependent mechanism may also contribute to butyrate-enhanced epithelial barrier function [49]. 

**Figure 4:**
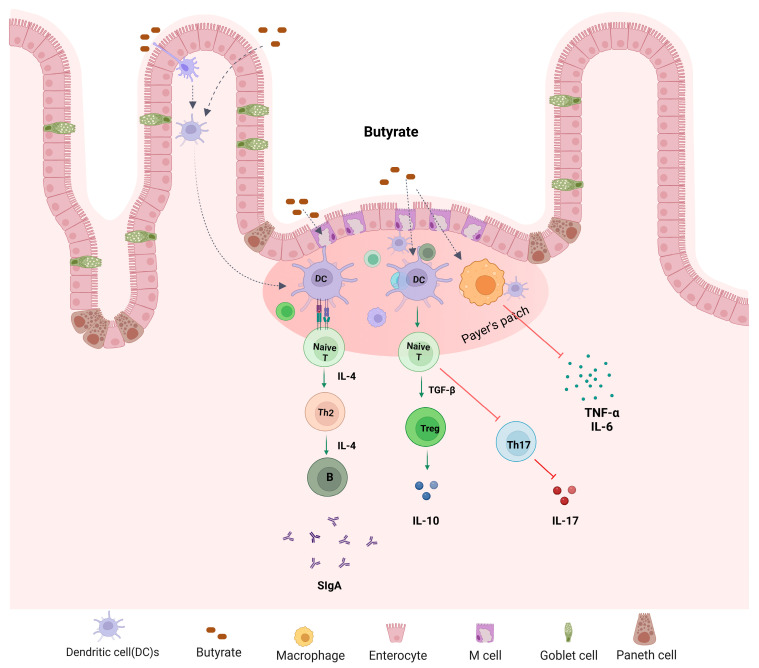
Microbial metabolite butyrate in the regulation of host immune function. Butyrate plays a key role in the maintenance of intestinal immune homeostasis. It promotes Treg differentiation and secretion of IL-10 and secretary IgA but inhibits Th17 cell differentiation.

An inappropriate immune response to antigens derived from intestinal components is a key feature in IBD, leading to an imbalance of inflammatory cytokines, tissue damage, and disease progression [50,51]. Increased phagocytic activity of macrophages and cytokines’ secretion (for example, IL-1, IL-6, IL-17, and TNF) has been found in IBD patients [52]. T lymphocytes (T-cells) play a crucial role in maintaining immune homeostasis by regulating innate and adaptive immune responses. Upon specific antigen stimulation, naïve CD4^+^ T-cells differentiate into effector T helper (Th) cells, including Th1, Th2, T regulatory (Treg), and Th17 cells [53]. Each Th type secretes specific cytokines to perform protective or pathogenic roles. Treg cells have immunosuppressive properties that help to maintain immune homeostasis by secreting anti-inflammatory cytokines, including IL-10 [54]. IBD is associated with dysregulated T-cell immune responses such as increased Th1, Th2, and Th17 cell function and decreased Treg cells function [55]. Th17 produces inflammatory cytokines such as IL-17A, IL-17F, and IL-21 which are involved in the pathogenesis of IBD. Gut microbial metabolite butyrate regulates the differentiation and proliferation of T cells (Figure 4). Butyrate administration enhanced Treg cell function and suppressed IL-17 levels as well as Th17 cells in the peripheral blood and colon tissues of TNBS-induced colitis rats compared to a control group [54]. Zimmerman et al. [56] have demonstrated that butyrate inhibits proliferation of both CD4^+^ and CD8^+^ T cells in a dose-dependent manner and it induces apoptosis in T cells through the Fas-mediated apoptosis pathway. Butyrate facilitates Treg cell differentiation by increasing histone H3 acetylation at the promoter and CNS3 region of the FOX3 gene locus [57]. Chen et al. [58] found that butyrate enhanced Th1 differentiation by promoting IFN-γ levels and T-bet expression in healthy conditions, but inhibited Th1 differentiation through IL-10 production and T-bet expression in colonic inflammation. In addition, butyrate has been shown to regulate inflammatory response by influencing NF-κB activity. NF-κB is a transcription factor involved in the regulation of various inflammatory mediators and cytokines expression including, TNF-α and IL-6 [59]. Butyrate is shown to reduce inflammatory response by suppressing NF-κB activity. Several studies have demonstrated the ability of butyrate to reduce NF-κB activity in human colon-cell lines and in lamina propria mononuclear cells isolated from CD patients [60,61,62]. Butyrate activates transmembrane GPRs and nuclear receptors such as aryl hydrocarbon receptor (AhR) in the intestinal epithelial cells. AhR is a ligand-activated transcription factor that resides in the cytosol in activated form, and translocates to the nucleus upon activation, thereby regulating AhR-dependent gene expression [63,64]. SCFAs, including butyrate, are shown to enhance AhR ligand interactions in mouse and human colon cells [44,65].

## 5. Therapeutic Implications of Butyrate for IBD

IBD is characterized by aberrant immune response and barrier dysfunction and is associated with a reduced number of butyrate-producing bacteria in the gut. As butyrate was found to not only provide energy to colonic epithelial cells but also help maintain intestinal integrity and modulate immune responses [43]; numerous studies have investigated the role of various forms of butyrate in reducing gut inflammation [66,67,68]. Many of the studies have demonstrated the efficacy of oral butyrate supplements, butyrate enema, butyrogenic diet, and bacterial supplements in the treatment of IBD.

### 5.1. Butyrate Supplements

#### 5.1.1. Oral Administration

Dysbiosis of gut microbiota leads to decreased butyrate synthesis and impaired butyrate metabolism as observed in IBD [69]. Although a low concentration of butyric acid is commonly present in our daily regular diet, it may not be sufficient to restore the epithelial function in inflammation in the colon. Many studies have investigated the therapeutic potential of butyrate oral supplements in gut inflammation in both preclinical studies and clinical trials. Table 2 summarizes these results (Table 2). Butyrate has been shown to reduce gut inflammation and ameliorate symptoms in a dose-dependent manner. Butyrate at 20 mg/kg/day or lower doses was found to have no significant effect, while at 100 mg/kg, it was effective against inflammation in mice [68,70]. Lee et al. [68] reported that the oral supplementation of sodium butyrate at 100 mg/kg of body weight daily decreased colitis scores, prevented body weight loss, and induced histone H3 acetylation in colonic mucosa in mouse models of acute and chronic colitis. Moreover, butyrate treatment restored the microbial community diversity and reduced microbiota dysbiosis in gut inflammation [71]. 

As orally supplemented butyrate is rapidly absorbed in the duodenum, the majority of the orally administered butyrate would not reach the colon. Moreover, the clinical application of oral butyrate is limited due to its unpleasant taste and odor. To address these issues, some studies used colon-targeted formulations and encapsulated butyrate to test if butyrate in such formulations has better effects in IBD patients, especially UC patients [67,72,73]. Sabatino et al. [72] demonstrated that enteric-coated butyrate tablets administration effectively reduced ileocaecal inflammation and maintained clinical remission in Crohn’s disease patients. Lipophilic microencapsulated sodium butyrate treatment showed enrichment of butyrogenic colonic bacteria in IBD patients [67]. Wang et al. [74] developed butyrate micelles so that butyrate is released in the lower gastrointestinal tract. They found that butyrate micelles significantly improved intestinal barrier function and reduced disease severity in DSS-induced colitis and CD45RB^hi^T-cell transfer colitis in mice.

**Table 2:** Impact of oral butyrate supplements on IBD.

	Treatment Name	Concentration	Colitis Model	Effects	Authors
Mice	Sodium butyrate	0.5% of sodium butyrate	DSS-induced colitis	Decreased mucosal inflammation	Vieira et al. [75]
Mice	Butyrate-releasing polysaccharide derivative	200 mg/kg	DSS-induced colitis	Reduced disease activity index, rebalanced gut microbiota, and reversed the imbalance between pro- and anti-inflammatory cytokines	Zha et al. [66]
Mice	Balatable butyrate-releasing derivative, *N*-(1-carbamoyl-2-phenylethyl) butyramide (FBA)	42.5 mg/kg	DSS-induced colitis	Reduced disease activity index	Simeoli et al. [76]
Mice	Sodium butyrate	200 mM	Citrobacter rodentium infection model	Prevented mice from weight loss and suppressed intestinal inflammation	Zhou et al. [77]
Mice	Sodium butyrate	200 mM	DSS-induced colitis	Suppressed intestinal inflammation and lowered pathology scores	Zhou et al. [77]
Mice	Sodium butyrate	5 g/L	TNBS induced colitis	Decreased disease activity index and suppressed inflammation	Chen et al. [78]
Mice	Sodium butyrate	100 mg/kg/day	DSS-induced acute colitis Piroxicam-induced chronic colitis	Decreased colitis scores and prevented weight loss	Lee et al. [68]
Mice	Sodium butyrate	N/A	DSS-induced colitis	Decreased disease activity index, and restored the balance of gut microbial communities	Dou et al. [71]
Mice	Sodium butyrate	150 mM	DSS-induced colitis	No significant difference in histologic scores	Lee et al. [79]
Human	Enteric-coated tablets	4 g/day	Crohn’s disease	Induced clinical improvement and reduced disease activity index	Sabatino et al. [72]
Human	Sodium butyrate tablets	4 g/day	Crohn’s disease	Induced clinical improvement or remission	Di Sabatino et al. [80]
Human	Microencapsulated sodium butyrate	1800 mg/day	IBD-both CD and UC	Increases the growth of bacteria able to produce SCFA with potential anti-inflammatory action	Faccin et al. [67]
Human	Microencapsulated sodium butyrate	1000 mg/day	UC in clinical remission	Helped to maintain clinical remission	Vernero et al. [73]
Human	Sodium butyrate	150 mg/twice a day	IBD-both CD and UC	No significant effects in newly diagnosed children and adolescents	Pietrzak et al. [81]

#### 5.1.2. Butyrate Enemas

Treatment with butyrate enemas had mixed effects in preclinical and clinical studies as summarized in Table 3. Butyrate enema showed inhibition of NF-κB activation in the lamina propria macrophages of UC patients, and it also reduced disease activity [82]. Segain et al. [60] observed a reduction of TNF-α induced NF-κB in colon tissues in butyrate enema-treated colitis rats. However, some clinical studies found that butyrate enema did not show any significant improvement in UC patients in remission and in patients with left-sided UC [83,84]. 

### 5.2. Butyrogenic Diets

As IBD is associated with decreased butyrate-producing bacteria and butyrate production in the colon; many investigators have tested if intake of butyrate-producing fermentable dietary fibers could be beneficial for IBD. Fernandez-Banares et al. [89] observed increased concentrations of fecal butyrate after the intake of fiber-rich *Plantago ovata* seeds in UC patients. Moreover, *Plantago ovata* seed supplementation showed effectiveness in maintaining UC remission. Further studies confirmed that butyrogenic diet supplementations attenuated colonic inflammation by the regulation of the gut microbial balance, increased production of SCFAs, upregulation of anti-inflammatory cytokines and Treg cells, and reduced mucosal damage (Table 4). A fiber-rich diet, such as oat bran and germinated barley foodstuff, has shown positive effects on IBD, especially in reducing the risk of relapse while maintaining prolonged remission in UC patients [90,91]. It was shown that β-glucan derived from oats and barley ameliorates colitis through the regulation of tight-junction proteins and inhibition of proinflammatory factors by increased SCFAs production via gut microbial fermentation [92,93]. IBD patients showed good tolerability to dietary-fiber intake, particularly during the clinical-remission stage [91,94]. Despite the beneficial effects of fiber, IBD patients are advised to reduce fiber consumption during the disease’s exacerbation period. Thus, the long-term effects of high fiber intake in active CD remain uncertain due to limited clinical data [95,96]. 

### 5.3. Combination Therapies

A combination therapy is a treatment modality that combines two or more therapeutic agents. It is found in most of the studies that combination therapies with butyrate and other agents are more effective than single therapy in the treatment of IBD or colitis models. Please see Table 5 for a summary of the outcomes of the studies (Table 5). Combinations of butyrate with other SCFAs, prebiotics, and probiotics have been investigated. A mixture of butyrate, *Pistacia atlantica*, and *Lactobacillus casei* or butyrate, *Lactobacillus casei*, and L-carnitin showed synergistic effects than a single agent in a TNBS-induced rat colitis model [102,103]. Combination of SCFAs, mainly acetate, propionate, and butyrate, showed increased effects against colitis [79]. However, treatment with a SCFAs rectal enema (sodium acetate, propionate, and butyrate) did not improve the histological and clinical state of left-sided UC [104]. Coadministration of sodium butyrate and mesalazine improved the efficacy of oral mesalazine in UC patients [105].

## 6. Discussion and Conclusions

Studies in the last two decades or so have shown that butyrate plays a critical role in the regulation of gut immune function and maintenance of barrier function and intestinal homeostasis. Butyrate regulates these functions by distinct transcriptional regulatory mechanisms, including inhibition of NF-κB and HDACs activation. The effects of butyrate on intestinal barrier function are in a dose-dependent manner, as high concentrations may induce apoptosis of epithelial cells and interrupt barrier function [41]. Most of the animal and human studies showed the positive effects of butyrate as a potential therapeutic agent to prevent inflammation and maintain remission in IBD. Butyrate oral supplements and butyrogenic diets are found to be effective in decreasing disease activity index and reducing inflammation. However, among nine preclinical studies on the effect of oral butyrate supplements in mouse models of colitis, one study showed no significant reduction in colon inflammation by butyrate supplement [79]. In that study, sodium butyrate was shown to modulate gut microbial composition compared to the control and colitis groups [79]. Similarly, in all four clinical studies, only one study reported no significant difference between sodium butyrate supplements [81]. Pietrzak et al. [81] assessed the effect of oral sodium butyrate along with standard therapy in newly diagnosed IBD children and adolescents and reported no significant effects comparing the sodium butyrate group with the placebo group. Butyrate administration is largely safe, though a few adverse effects have been noted [107]. Lin et al. [107] noticed that butyrate at excessive doses (more than 150 mmol/L) induced minimal mucosal damage in the colon and distal ileum in newborn rats.

A limitation of this review is that we did not perform any statistical analysis such as meta-analysis for the included studies. This is mainly due to the scarcity of data and heterogeneity of the studies, as various doses, forms, and administration routes of butyrate have been used in these studies. More clinical trials are required to determine the effective doses and forms of butyrate supplements for IBD patients.

In conclusion, butyrate at appropriate concentrations helps to maintain intestinal barrier function and regulate the immune response in the gut. Clinical trials and animal studies have shown that butyrate can reduce mucosal inflammation and improve barrier function in UC and CD. Butyrate formulations and butyrogenic compounds may represent alternative therapeutic approaches for IBD. Combination therapies with butyrate and other SCFAs may further increase the efficacy of butyrate in the treatment of IBD. Although most of the studies have shown the beneficial effects of butyrate in colitis models and IBD patients, more clinical studies are needed to understand the impact of butyrate administration alone or with standard therapy in the management of IBD.

## Figures and Tables

**Table 1:** Comparison of Crohn’s disease and ulcerative colitis.

Features	Crohn’s Disease	Ulcerative Colitis
Location	Any part of the GI * tract	Large intestine
Inflammation	Transmural	Superficial
Complications	Fistula development, obstruction	No fistula, Hemorrhage
Distribution	Discontinuous and patchy	Continuous
Rectal involvement	Occasional	Common

* GI: gastrointestinal.

**Table 3:** Impact of butyrate enemas on IBD.

	Concentration	Colitis Model	Duration	Effects	Authors
Rat	3 mL of 100 mM	DSS-induced colitis	17 days	Decreased ulcer index and myeloperoxidase activity	Okamoto et al. [85]
Rat	100 mM sodium butyrate	TNBS-induced colitis	Day 5 to 23	Decreased inflammation and improved clinical recovery	Butzner et al. [86]
Rat	3% of sodium butyrate	DSS-induced colitis	N/A	Decreased mucosal damage, no difference in the incidence of diarrhea	Kanauchi et al. [87]
Rat	100 mM sodium butyrate	TNBS-induced colitis	2 weeks	Decreased inflammation and stimulated mucosal repair	Segain et al. [60]
Human	100 mM sodium butyrate	Ulcerative colitis	2 weeks	Decreased disease activity index and inflammation	Scheppach et al. [88]
Human	60 mL of 80 mM sodium butyrate	Ulcerative colitis	3 and 6 weeks	Nightly butyrate enema was not efficacious for distal ulcerative colitis	Steinhart et al. [84]
Human	60 mL of 100 mM sodium butyrate	Ulcerative colitis	4 and 8 weeks	Decreased disease activity index and mucosal inflammation after 8 weeks	Luhrs et al. [82]
Human	60 mL of 100 mM sodium butyrate	Ulcerative colitis	20 days	No significant effects of butyrate administration on parameters of oxidative stress were found	Hamer et al. [83]

**Table 4:** Impact of butyrogenic diet on IBD.

	Treatment	Disease or Model	Effects	Authors
Rat	Germinated barley foodstuff	DSS-induced colitis	Bloody diarrhea and mucosal damage were dose dependently decreased	Kanauchi et al. [87]
Mice	Flaxseed oligosaccharides	DSS-induced colitis	Decreased disease activity index, improved colon histology, and increased cecal SCFAs levels	Xu et al. [97]
Mice	Oat β-glucan	DSS-induced colitis	Suppressed colonic inflammatory infiltration and increased SCFAs concentrations	Bai et al. [93]
Mice	Butyl-fructooligosaccharides	DSS-induced colitis	Increased cecal butyrate concentration, increased occludin mRNA expression	Kang et al. [98]
Mice	Soluble dietary fiber from quinoa bran	DSS-induced colitis	Decreased disease activity index, increased microbial diversity and SCFAs	Liu et al. [99]
Mice	Peanut skin procyanidins extract	DSS-induced colitis	Suppressed inflammatory responses, increased butyrate producing bacterial abundance, and colon SCFAs	Wang et al. [100]
Human	*Plantago ovata* seeds	Ulcerative colitis in remission	Increased fecal butyrate levels	Fernandez-Banares et al. [89]
Human	Oat bran	Ulcerative colitis	Increased fecal butyrate and maintained the remission phase	Hallert et al. [90]
Human	Germinated barley foodstuff	Ulcerative colitis in remission	Effective in the maintenance of prolonged remission	Hanai et al. [91]
Human	Prebiotic oligofructose-enriched inulin	Crohn’s disease	The relative levels of butyrate and acetaldehyde increased compared to the baseline	De Preter et al. [101]
Human	Oat bran	Ulcerative colitis in remission	Increased fecal SCFAs, including butyric acid, and reduced the risk of relapse	Nyman et al. [94]

**Table 5:** Effects of butyrate combination therapies on IBD.

	Treatment Name	Concentration	Colitis Model	Duration	Effects	Authors
Mice	SCFAs	67.5 mM acetate, 40 mM butyrate, 25.9 mM propionate	DSS-induced colitis	N/A	No significant difference in histologic scores but IL-17A producing T cells increased	Lee et al. [79]
Rat	*Pistacia atlantica*, butyrate, *Lactobacillus casei*	25 mg/kg *atlantica*, 0.5% butyrate, and 108 CFU of *Lactbacillus*	TNBS-induced colitis	10 days	Reduced the severity of inflammation	Gholami et al. [102]
Human	*Plantago ovata* seeds and mesalamine	20 g seeds and 1.5 g mesalamine/day	Ulcerative colitis remission	12 months	Effective in remission maintenance	Fernandez-Banares et al. [89]
Human	Sodium butyrate and mesalazine	4 g/day butyrate and 2.4 g/day mesalamine	Ulcerative colitis	6 weeks	Improved the efficacy of mesalazine	Vernia et al. [105]
Human	Calcium magnesium butyrate along with Mezavant treatment	1.2 g/day magnesium butyrate and 4.8 g/day mezavant	Ulcerative colitis	N/A	Relief of symptoms	Gibbs and Brown. [106]

## Data Availability

Available upon request.
